# Dermatomyositis sine myositis – Case presentation

**DOI:** 10.31138/mjr.28.1.57

**Published:** 2017-03-28

**Authors:** Evripidis Kaltsonoudis, Eleftherios Pelechas, Alexandros A. Drosos

**Affiliations:** Rheumatology Clinic, Department of Internal Medicine, Medical School University of Ioannina, Ioannina, Greece

**Keywords:** Dermatomyositis sine myositis, heliotrope rash, Gottron’s papules

## Abstract

In this case, we present a patient with unilateral salivary gland enlargement and periorbital edema with erythematous rash. We discuss the differential diagnosis and the relevant therapy.

## CASE PRESENTATION

A 45-year-old Caucasian female presented to her family doctor complaining about left salivary gland swelling, edema and rash on both eyelids and cheeks. Physical examination did not show any other findings and the patient denied any additional symptoms. Patient’s family and personal history were of no significance.

At that time, a routine laboratory workup, including full blood count (FBC) – urea and electrolytes (U&E) – liver function tests (LFTs) – muscle enzymes – inflammatory markers, did not reveal any significant abnormalities. A chest x-ray, tuberculin skin test and an abdominal ultrasound were ordered, but did not show any particular findings either. Further immunological testing showed anti-nuclear antibodies (ANA) at a titer of 1/160 speckled. No other autoantibodies were detected. A workup for dry eyes and mouth with Schirmer’s test I (11mm), Rose Bengal and salivary gland biopsy were negative.

A private doctor made a diagnosis of Sjögren’s syndrome arbitrarily and administered hydroxychloroquine 200mg twice a day plus prednisolone 10mg once a day. This therapeutic approach was not beneficial to the patient.

**Figure 1:**
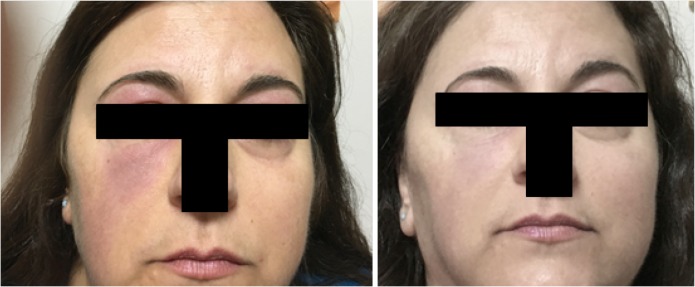
**Left** – Edema and violaceous rash on both eyelids, left salivary gland enlargement. **Right** – Improvement of the skin rash after treatment with high dose of methylprednisolone.

Three months later, the patient visited the Rheumatology outpatient clinic, because of a persistent facial erythema and periorbital violaceous discoloration. After a thorough physical examination in our clinic, there were no other findings except the heliotrope rash of the eyelids, an asymmetrical face erythema and the periorbital edema (**Figure 1 - left**). The family medical history was of no significant importance. Personal medical history revealed photosensitivity but no other significant pathologies. She denied muscle weakness, vision disorders or pain in the eyes, morning stiffness, oral ulcers or dry mouth, loss of appetite or weight. Electromyography did not show any abnormal findings and the values of a new laboratory workup were within normal limits.

Thus, the objective findings were a bilateral periorbital edema, face erythema, heliotrope-like rash on both eyelids, ANA titer of 1/160 speckled and the subjective photosensitivity.

The differential diagnosis includes:

1. Inflammatory dermatological disorders or drug-induced disorders;
2. Sjögren’s syndrome (SS);
3. Systemic Lupus Erythematosus (SLE); and
4. Dermatomyositis sine myositis (DsM).

1. Allergic or drug-induced dermatological disorders could be a possible diagnosis.^1^ A simple question regarding the current or past medication can safely count out drug-induced dermatological disorders. Allergic reactions could appear with eyelid edema and a rash^2^ but usually they are self-limiting and they disappear after the administration of prednisone.
2. Although Sjögren’s syndrome could manifest with parotid gland enlargement (unilateral or bilateral) as the first manifestation,^3^ in this case, the patient did not have xerostomia, xerophthalmia and also had a negative Schirmer’s test and a negative salivary gland biopsy. Finally, the laboratory workup did not show any specific autoantibodies.
3. In order to diagnose SLE, 4 out of 11 criteria should be met.^4^ In our case, the patient had (subjective) photosensitivity, and a (weakly) positive ANA titer only two of those criteria. Patients with Dermatomyositis are at times difficult to distinguish from patients with subacute cutaneous lupus erythematosus.^5^
4. Dermatomyositis sine myositis or amyopathic Dermatomyositis is a rare but distinct subtype of Dermatomyositis.^6^ It is diagnosed in patients with typical cutaneous manifestations (consisting of heliotrope rash, facial erythema and edema, Gottron’s papules and periungual telangiectasia) in whom there is no evidence of muscle weakness and who repeatedly have normal serum muscle enzyme levels. There is a female to male preponderance (3:1) and the onset of the disease usually occurs in early adulthood. Dermatomyositis sine myositis should be aggressively treated even in the absence of muscle involvement since intense and prolonged skin inflammation can result in cutaneous ulceration and calcinosis. Treatment is based on systemic immunosuppressive therapy (high dose corticosteroids, methotrexate, azathioprine, mycophenolate) or immunomodulatory therapy (high dose intravenous immunoglobulins).

In the presented case, the patient was treated with high dose of methylprednisolone (32mg) once a day along with calcium and vitamin D supplements. A month later, the patient showed significant improvement of the rash **(Figure 1 - right)**. In addition, she did not develop muscle weakness or muscle enzyme abnormalities.

In conclusion, DsM, even a rare condition, should be a differential to be borne in mind for clinicians because it needs an aggressive treatment in order to prevent chronic skin changes and systemic complications such as pulmonary involvement. Finally, a close observation of the patient is mandatory, as DsM has been associated with different types of malignancies.

## References

[1] Bachot N Roujeau J C Differential diagnosis of severe cutaneous drug eruptions. Am J Clin Dermatol. 2003;4:561–72. 12862499 10.2165/00128071-200304080-00006

[2] Goossens A Contact allergic reactions on the eyes and eyelids. Bull Soc Belge Ophtalmol. 2004;292:11–8. 15253485

[3] Spijkervet F K Haacke E Kroese F G Bootsma H Vissink A Parotid Gland Biopsy, the Alternative Way to Diagnose Sjögren Syndrome. Rheum Dis Clin North Am 2016;42:485–99. 27431350 10.1016/j.rdc.2016.03.007

[4] Aringer M Dörner T Leuchten N Johnson S R Toward new criteria for systemic lupus erythematosus-a standpoint. Lupus 2016;25:805–11. 27252256 10.1177/0961203316644338

[5] Fett N M Fiorentino D Werth V P Practice and Educational Gaps in Lupus, Dermatomyositis, and Morphea. Dermatol Clin. 2016;34:243–50. 27363879 10.1016/j.det.2016.02.006 PMC5514844

[6] Dalakas M C Hohlfeld R Polymyositis and dermatomyositis. Lancet 2003;362:971–82. 14511932 10.1016/S0140-6736(03)14368-1

